# Human Embryonic Stem Cells Acquire Responsiveness to TRAIL upon Exposure to Cisplatin

**DOI:** 10.1155/2019/4279481

**Published:** 2019-01-21

**Authors:** Lucie Pešková, Vladimír Vinarský, Tomáš Bárta, Aleš Hampl

**Affiliations:** ^1^Department of Histology and Embryology, Faculty of Medicine, Masaryk University, Kamenice 5, 62500 Brno, Czech Republic; ^2^International Clinical Research Center, St. Anne's University Hospital, Pekařská 53, 656 91 Brno, Czech Republic

## Abstract

Tumor necrosis factor-related apoptosis-inducing ligand—TRAIL—is a protein operating as a ligand capable of inducing apoptosis particularly in cancerously transformed cells, while normal healthy cells are typically nonresponsive. We have previously demonstrated that pluripotent human embryonic stem cells (hESC) are also refractory to TRAIL, even though they express all canonical components of the death receptor-induced apoptosis pathway. In this study, we have examined a capacity of DNA damage to provoke sensitivity of hESC to TRAIL. The extent of DNA damage, behavior of molecules involved in apoptosis, and response of hESC to TRAIL were investigated. The exposure of hESC to 1 *μ*M and 2 *μ*M concentrations of cisplatin have led to the formation of 53BP1 and *γ*H2AX foci, indicating the presence of double-strand breaks in DNA, without affecting the expression of proteins contributing to mitochondrial membrane integrity. Interestingly, cisplatin upregulated critical components of the extrinsic apoptotic pathway—initiator caspase 8, effector caspase 3, and the cell death receptors. The observed increase of expression of the extrinsic apoptotic pathway components was sufficient to sensitize hESC to TRAIL-induced apoptosis; immense cell dying accompanied by enhanced PARP cleavage, processing of caspase 8, and full activation of caspase 3 were all observed after the treatment combining cisplatin and TRAIL. Finally, we have demonstrated the central role of caspase 8 in this process, since its downregulation abrogated the sensitizing effect of cisplatin.

## 1. Introduction

Human embryonic stem cells (hESC), derivatives of the inner cell mass of the human blastocyst, are a unique cell type that offers a variety of applications, spanning from the modelling of diseases to the development of strategies for regenerative medicine. It is well established that cultured hESC undergo apoptosis upon various stress stimuli [1–4]. By default, hESC activate intrinsic (mitochondrial) apoptotic signaling but not much is known about extrinsic (receptor-activated) apoptosis in these cells [5–7]. We have previously shown that even though hESC express corresponding receptors on their surface and are molecularly fully equipped to activate extrinsic apoptotic signaling pathways, they are still refractory to tumor necrosis factor-related apoptosis-inducing ligand (TRAIL) [8]. TRAIL is a member of the tumor necrosis factor (TNF) family that came into the spotlight as a potential antitumor agent, as it is capable of inducing apoptosis selectively in transformed/cancer cells [9, 10]. TRAIL forms homotrimers and triggers the extrinsic apoptotic pathway upon binding to receptors presented on the cell surface [9]. There are five known TRAIL receptors: (i) death receptor DR4 (TRAILR1); (ii) death receptor DR5 (TRAILR2)—both DR4 and DR5 contain the intracellular death domain (DD) allowing the transmission of the apoptotic signal; (iii) decoy receptor DcR1 (TRAILR3)—lacking DD; (iv) decoy receptor DcR2 (TRAILR4)—containing truncated DD; and (v) soluble receptor osteoprotegerin (TRAILR5)—not capable of transmitting the apoptotic signal [11]. TRAIL-induced activation of death receptors leads to receptor oligomerization and subsequently to the recruitment of the Fas-associated DD protein (FADD) and procaspase 8 or procaspase 10, finally resulting in the formation of the death-inducing signaling complex (DISC). Upon DISC formation, procaspase 8 is autocatalytically activated leading to the cleavage and activation of caspase 3, which then in turn cleaves a number of intracellular targets including poly (ADP-ribose) polymerase (PARP), a hallmark of ongoing apoptosis [12, 13]. The apoptotic signal induced by TRAIL can be blocked at the level of DISC formation by the cellular FLICE-inhibitory protein (cFLIP). This inactive homolog of caspase 8 inhibits the transmission of the apoptotic signal by forming the apoptosis inhibitory complex prior to DISC formation [14].

As announced above, TRAIL is capable of apoptosis induction in transformed/cancer cells, while normal differentiated and/or progenitor cells are nonresponsive to this molecule. We have previously demonstrated that undifferentiated hESC also belong to TRAIL-refractory cells [8]. This resistance is mainly due to high levels of cFLIP but can be overcome by sensitizing hESC by proteosynthesis inhibition. In the current study, we have examined whether hESC can also be sensitized to TRAIL by exposure to the DNA-damaging compound cisplatin. We demonstrate that induction of temporary reparable DNA damage makes the cells responsive to TRAIL. We also show that this responsiveness to TRAIL is mainly due to an increased expression of caspase 8, which stands out as a crucial mediator of extrinsic apoptotic signals in hESC.

## 2. Materials and Methods

### 2.1. Culture and Treatment of hESC

Two independent lines of hESC (CCTL12 and CCTL14) [15] (passage numbers 30-65) were cultured on Matrigel- (BD Biosciences) coated dishes in mouse embryonic fibroblast- (MEF-) conditioned hESC medium (CM). CM was prepared by the incubation of the hESC medium (DMEM/F12 supplemented with 15% Knockout Serum Replacement, 2 mM L-glutamine (Invitrogen, Life Technologies Ltd.), 1x MEM nonessential amino acid solution, 1x penicillin/streptomycin (PAA Laboratories GmbH, Pasching, Austria), *β*-mercaptoethanol (Sigma-Aldrich), and 4 ng/ml human FGF-2 (PeproTech Inc., Rocky Hill, NY, USA)) with mitotically inactivated mouse embryonic fibroblasts (MEF). After 24 hours, CM was collected, supplemented with 2 mM L-glutamine (Invitrogen) and 10 ng/ml FGF-2 (PeproTech Inc.), and filtered. Cells were cultured in a humidified incubator at 37°C in 5% CO_2_ atmosphere and passaged every 3–7 days using TrypLE™ Express (Gibco).

For cisplatin treatment, hESC were allowed to grow for 2 days after passage and then treated with cisplatin (Sigma-Aldrich) for 24 hours. For the sensitization experiment, hESC were cultured for an additional 24 hours in CM containing 200 ng/ml TRAIL (Apronex). For chemical inhibition of caspase 10, hESC grew 2 days after passage and then they were treated with 2 *μ*M cisplatin. After 24 hours, synthetic caspase 10 inhibitor Z-AEVD-fmk (Abcam) at 20 *μ*M concentration together with 200 ng/ml TRAIL were added for either 12 or 24 hours.

### 2.2. Flow Cytometric Quantification of Apoptosis

An extension of apoptosis was determined using an Annexin-V-FLUOS Staining Kit (Roche) according to the manufacturer's instructions. Briefly, both adherent and detached cells were collected, washed with PBS, and incubated in labeling solution containing Annexin-V-FLUOS and propidium iodide for 15 minutes at room temperature in the dark. Flow cytometry was performed using Cytomics FC500 (Beckman Coulter) and the data were analyzed with FlowJo 7.2.2 software (http://www.flowjo.com).

### 2.3. Immunocytochemistry

Cells growing on coverslips coated with Matrigel (BD Biosciences) were washed with phosphate-buffered saline (PBS) (pH 7.4) and fixed in 4% paraformaldehyde for 15 minutes at room temperature (RT). After fixation, cells were washed three times with PBS, permeabilized with 0.1% Triton X-100 (Sigma-Aldrich) in PBS at RT for 10 minutes, and washed three times with PBS. Cells were incubated with primary antibodies (phosphorylated H2AX at serine 139 (#05-636, Millipore) and 53BP1 (#4937, Cell Signaling Technology)) for 1 hour at RT. Cells were washed three times with 0.03% Tween (Sigma-Aldrich) in PBS and incubated with secondary antibodies (Alexa Fluor 488 (#A11008) and Alexa Fluor 594 (#A11058) (Invitrogen)) for 1 hour at RT in the dark. Nuclei were stained with 4′,6-diamidino-2-phenylindole (DAPI) (Sigma-Aldrich) and washed 5 times with 0.03% Tween in PBS, and then cells were mounted in Mowiol containing 1,4-diazobicyclo(2.2.2.)-octane to prevent fading. Microscopy was performed using an Olympus Cell-R microscope (Olympus C&S Ltd., Prague, Czech Republic, https://www.olympus-global.com/).

### 2.4. Western Blotting

Cells were washed three times with PBS and lysed in buffer containing 50 mM Tris-HCl (pH 6.8), 1% sodium dodecyl sulfate (SDS), and 10% glycerol. Protein concentration was determined using the DC Protein Assay (Bio-Rad) and adjusted to 1 mg/ml. Lysates were supplemented with bromophenol blue (0.01%) and *β*-mercaptoethanol (1%) and subjected to 100°C for 5 minutes. Cell lysates were separated by SDS-polyacrylamide gel electrophoresis and transferred to a polyvinylidene difluoride membrane (PVDF) (Millipore). Membranes were blocked in 5% low fat milk in TRIS-buffered saline with Tween (TBST) (pH 7.6) and incubated with a primary antibody (4°C, overnight) and then with a secondary antibody (RT, 1 hour). For visualization, an ECL Plus reagent kit (GE Healthcare) was used. The following primary antibodies were used: Bcl-xL (#2764), Bax (#2772), caspase 3 (#9662), caspase 8 (#9746), cleaved PARP (#5625) (all from Cell Signaling Technology), p53 (DO-1 clone, kindly provided by Dr. Bořivoj Vojtěšek (Regional Centre for Applied Molecular Oncology, Masaryk Memorial Cancer Institute, Brno)), XIAP (48-hILP-XIAP, BD Transduction Laboratories), Mcl-1 (M8434, Sigma-Aldrich), and caspase 10 (#M059-3, MBL International Corporation). To demonstrate the uniformity of protein loading, PVDF membranes were stained with 0.1% amidoblack.

### 2.5. Flow Cytometric Analysis of Death Receptor Expression

Cells were harvested, washed with PBS, and incubated in cold PBS containing 20% human AB serum (Faculty Hospital, Brno) and 0.2% fish gelatin (G7765, Sigma-Aldrich) for 10 minutes on ice. Cells were then washed in PBS-G buffer (PBS + 0.2% fish gelatin + 0.1% NaN_3_) and incubated with a primary antibody for 1 hour on ice. The following primary antibodies were used: DR4 (DR-4-02, EXBIO), DR5 (DR5-01-1, EXBIO), DcR1 (HS301, Enzo Life Sciences), and DcR2 (HS402, Enzo Life Sciences). Cells were then washed twice in PBS-G buffer, incubated with the R-phycoerythrin-conjugated secondary antibody (1070-09, Southern Biotech) for 30 minutes on ice, and washed twice with PBS-G buffer. Death receptor expression was measured using the Cytomics FC500 flow cytometer (Beckman Coulter) and analyzed with FlowJo 7.2.2 software (http://www.flowjo.com).

### 2.6. Flow Cytometric Analysis of Surface Markers of Pluripotency

Cells were harvested, washed with ice-cold wash buffer (PBS with 10% fetal bovine serum), and incubated with a primary antibody (SSEA-3 and TRA-1-81, kindly provided by Prof. Peter W. Andrews (Department of Biomedical Science, University of Sheffield)) on ice in the dark for 30 minutes. Cells were washed twice in wash buffer, incubated with the R-phycoerythrin-conjugated secondary antibody (1070-09, Southern Biotech) in the dark for 30 minutes and then washed twice with wash buffer. Flow cytometry was performed using Cytomics FC500 (Beckman Coulter) and the results were analyzed with FlowJo 7.2.2 software (http://www.flowjo.com).

### 2.7. Downregulation of Caspase 8

For caspase 8 downregulation experiments, hESC were transfected with siRNA using a Neon transfection instrument (Invitrogen) according to the manufacturer's instructions. 100 pmoles of caspase 8 siRNA (Santa Cruz Biotechnology Inc.) were added to 1 × 10^5^ cells and transfection was performed with one 1100 V pulse for 30 ms. Cells were seeded on Matrigel and cultured in CM without antibiotics. Cells were harvested at 48 and 72 hours after transfection.

## 3. Results

### 3.1. Determination of Concentration of Cisplatin That Causes Reparable DNA Damage in Cultured hESC

**Figure 1:**
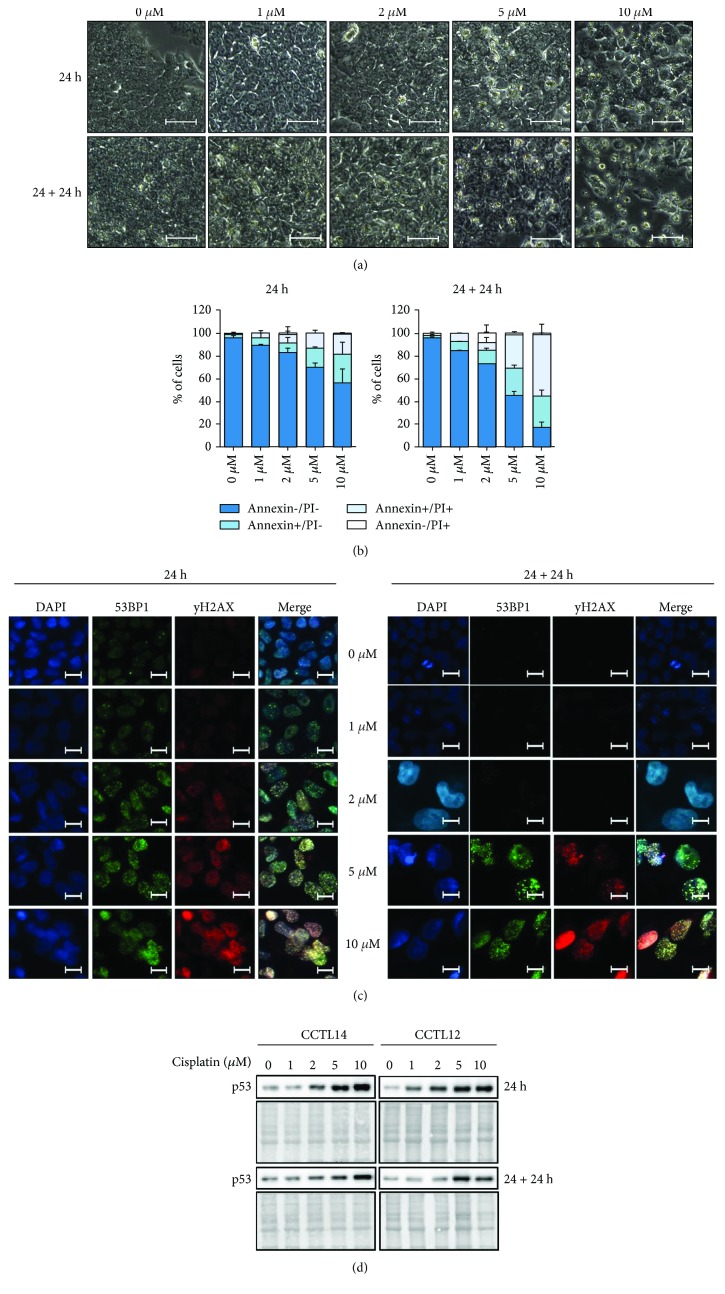
Cisplatin at concentrations of 1 *μ*M and 2 *μ*M induces DNA damage but not excessive apoptosis in hESC. Cells were treated with cisplatin at concentrations ranging from 1 *μ*M to10 *μ*M for 24 hours and then either harvested and fixed (24 h) or further cultured in the absence of cisplatin for 24 hours (24 + 24 h). (a) Morphology of nontreated and cisplatin-treated hESC as observed by light microscopy (10x, bar 50 *μ*m). Both CCTL12 and CCTL14 lines of hESC were used (*n* = 1 for CCTL12; *n* = 3 for CCTL14); a representative picture is shown. (b) Graphs showing cell death incidence as determined by flow cytometric analysis after double staining with Annexin-V and propidium iodide. At least 10,000 cells were analyzed per sample. Living (Annexin-/PI-), apoptotic (Annexin+/PI-), necrotic (Annexin-/PI+), and secondary necrotic cells (Annexin+/PI+) are reported as a percentage of the total cell count. The CCTL14 line of hESC was used (*n* = 2). (c) The presence of double-strand breaks in DNA as visualized by 53BP1 and *γ*H2AX foci using indirect immunofluorescence (40x, bar 20 *μ*m). Both CCTL12 and CCTL14 lines of hESC were used (*n* = 2); a representative picture is shown. (d) The quantity of p53 protein in cisplatin-treated hESC as demonstrated by western blot analysis. A PVDF membrane stained with 0.1% amidoblack was used as a loading control. Both CCTL12 and CCTL14 lines of hESC were used (*n* = 2).

Cisplatin is a widely used anticancer drug that binds covalently to purine bases in the major groove of DNA, resulting in DNA crosslinking and to subsequent formation of double-strand breaks (DSB). The aim here was to apply cisplatin at the dose that (i) induces DNA damage in hESC, (ii) enables repair of DNA upon cisplatin withdrawal, and (iii) does not lead to the death of more than 50% of the cell population upon cisplatin withdrawal. To determine such concentration, we have exposed hESC for 24 hours to cisplatin at 1, 2, 5, and 10 *μ*M concentrations, respectively, and then we cultured the cells for a further 24 hours to evaluate its effects. As shown in Figure 1(a), exposure to cisplatin has led to changes in cell morphology—cells treated with cisplatin appeared larger when compared to untreated controls, with these changes being more prominent in higher cisplatin concentrations. This alteration to cell morphology was also unraveled by flow cytometric analysis as a shift in side scatter parameters (Supplementary Figure 1(a)). When determined immediately after 24-hour exposure to cisplatin, the percentage of dying cells (costained with Annexin-V and propidium iodide) was less than 50% for all concentrations of the drug. In contrast, 24 hours after withdrawal from cisplatin, the cells exposed to higher concentrations (5 *μ*M and 10 *μ*M) suffered from massive dying (55.13 ± 4.03% and 82.88 ± 4.71% of dead cells, respectively), while a much smaller proportion of cells (15.32 ± 0.18% and 26.92 ± 1.93%, respectively) died upon the exposure to lower concentrations of the drug (1 *μ*M and 2 *μ*M) (Figure 1(b); Supplementary Figure 1(b)). To evaluate an association of cell dying (ability of cells to recover) with the level of damage to their DNA, we then visualized the p53-binding protein (53BP1) and phosphorylated histone H2AX at serine 139 (*γ*H2AX). These molecules are recruited to DNA lesions and form foci, which are detectable using immunofluorescence [16, 17]. The number of 53BP1- and *γ*H2AX-positive cells as well as the level of DNA damage demonstrated by the number of foci were both gradually increased in a dose-dependent manner, when determined at the time of withdrawal of cisplatin. Importantly, when cultured for an additional 24 hours in the absence of cisplatin, cells treated with 1 *μ*M and 2 *μ*M of cisplatin recovered and completely repaired their DNA, as demonstrated by the absence of 53BP1 and *γ*H2AX foci, which was not the case for higher concentrations (Figure 1(c)). To confirm the recovery of hESC from DNA damage, we have also measured at the same two time points the levels of p53. As shown in Figure 1(d), the p53 protein increased its levels in hESC upon treatment with all four doses of cisplatin. Twenty four hours after cisplatin withdrawal, hESC exposed to 1 *μ*M and 2 *μ*M doses of cisplatin downregulated the levels of p53, while hESC exposed to higher concentrations maintained elevated p53 levels. Taken together, these three sets of data collectively demonstrated that cisplatin at concentrations of 1 *μ*M and 2 *μ*M produced reversible effects, so that it can be used at these concentrations for sensitizing hESC.

### 3.2. Sensitizing of hESC towards Apoptosis Induced by TRAIL

**Figure 2:**
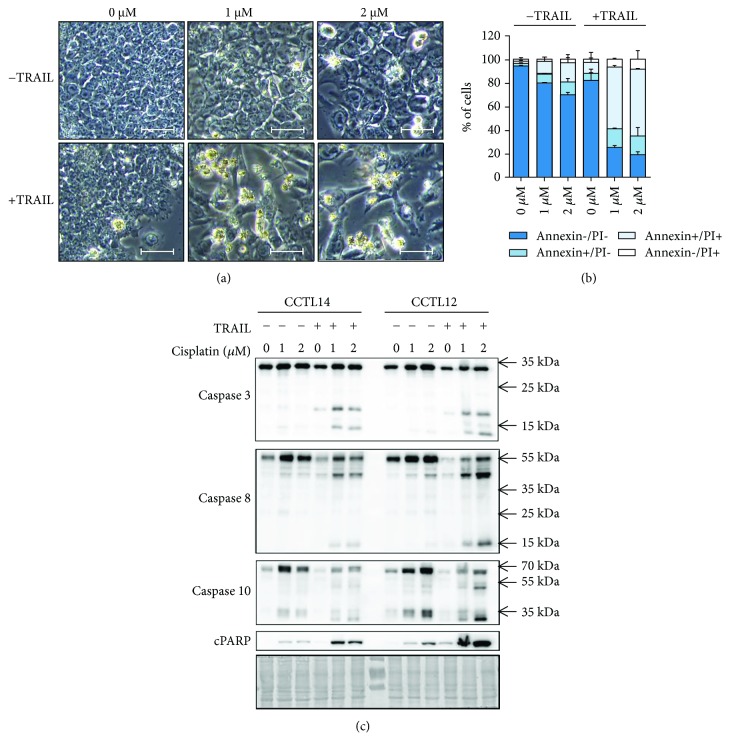
Cisplatin sensitizes hESC towards TRAIL-induced apoptosis. Cells were cultured in the presence of 1 *μ*M and 2 *μ*M doses of cisplatin for 24 hours and then they were treated with recombinant human TRAIL (200 ng/ml) for 24 hours. (a) Morphology of hESC upon the given treatments as observed by light microscopy (10x, bar 50 *μ*m). Both CCTL12 and CCTL14 lines of hESC were used (*n* = 1 for CCTL12; *n* = 3 for CCTL14); a representative picture is shown. (b) Graphs showing cell death incidence as determined by flow cytometric analysis after double staining with Annexin-V and propidium iodide. At least 10,000 cells were analyzed per sample. Living (Annexin-/PI-), apoptotic (Annexin+/PI-), necrotic (Annexin-/PI+), and secondary necrotic cells (Annexin+/PI+) are reported as a percentage of the total cell count. The CCTL14 line of hESC was used (*n* = 2). (c) Activation of the caspase cascade and PARP cleavage upon the given treatments as determined by western blot. Inactive full length caspases include procaspase 3 (~35 kDa), procaspase 8 (~55 kDa), and procaspase 10 (~60 kDa); their active forms include cleaved caspase 3 (~17/12 kDa), cleaved caspase 8 (~18 kDa), and cleaved caspase 10 (~20 kDa). Staining with 0.1% amidoblack was used to evaluate the protein loading. Both CCTL12 and CCTL14 lines of hESC were used (*n* = 2).

To initially determine whether cisplatin sensitizes hESC towards TRAIL-induced apoptosis, we first exposed the cells for 24 hours to 1 *μ*M and 2 *μ*M doses of cisplatin, respectively. Immediately after withdrawal of cisplatin, the cells were either treated for an additional 24 hours with TRAIL or kept in a medium without TRAIL. We used TRAIL at a concentration of 200 ng/ml, which has been previously shown to induce massive apoptosis in TRAIL-sensitive cell lines but not in hESC [8, 18]. As shown in Figure 2(a), the changes to hESC morphology were much more pronounced when TRAIL was combined with cisplatin, compared to TRAIL and/or cisplatin treatments alone (Figure 2(a), Supplementary Figure 2(a)). While TRAIL alone induced the cell death of 17.52 ± 10.21% cells, combining TRAIL with 1 *μ*M and/or 2 *μ*M doses of cisplatin have led to the death of 74.82 ± 1.24% and 80.79 ± 2.01% cells, respectively (Figure 2(b), Supplementary Figure 2(b)). Corresponding with the occurrence of cell dying, hESC contained activated caspases 3 and 8, and also cleaved PARP (cPARP), together confirming ongoing apoptosis (Figure 2(c)). It is notable that both drugs induced detectable processing of initiator caspase 8 and effector caspase 3 into its intermediates also when they were used separately (Figure 2(c)). Still, the full activation of caspase 8 (as determined by the presence of a 18 kDa fragment) and caspase 3 (as determined by its active 17/12 kDa fragments) was achieved only when cisplatin and TRAIL were combined. Together, this set of data demonstrates that hESC gain sensitivity to TRAIL when exposed to cisplatin.

### 3.3. Changes in Apoptotic Molecular Pathway That Are Associated with Sensitizing of hESC by Cisplatin

**Figure 3:**
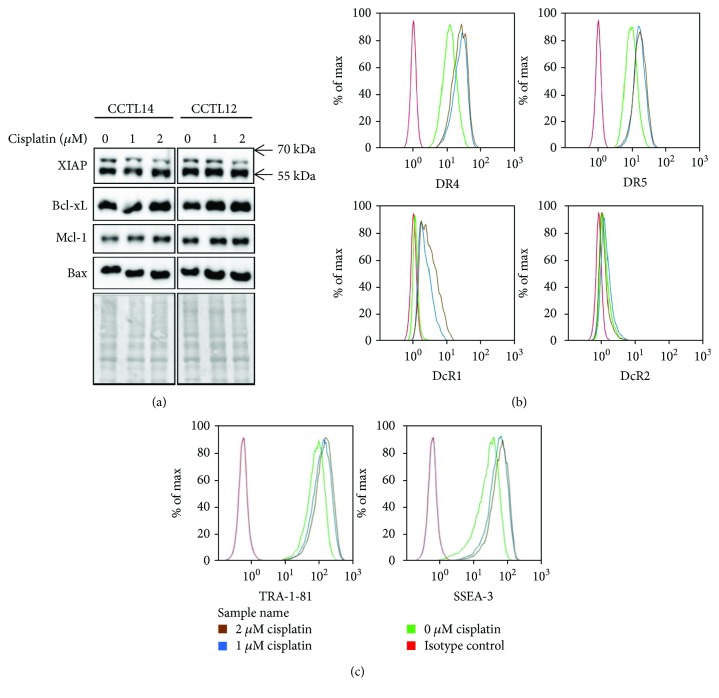
A sublethal dose of cisplatin modulates the expression of death receptors and initiator caspases. Cells treated with 1 *μ*M and 2 *μ*M doses of cisplatin for 24 hours were assessed for the expression of key apoptotic signaling components and pluripotency-associated markers. (a) Western blot analysis of pro- (Bax) and antiapoptotic proteins (XIAP, Bcl-xL, and Mcl-1) involved in the regulation of the intrinsic apoptotic pathway. Staining with 0.1% amidoblack was used to evaluate the protein loading. Both CCTL12 and CCTL14 lines of hESC were used (*n* = 2). (b) Expression of cell membrane-linked death receptors with an affinity to TRAIL and (c) expression of pluripotency-associated membrane markers, as determined by flow cytometry. At least 10,000 cells were analyzed per sample. Red line—isotype control, green line—control cells without cisplatin, blue line—cells treated with 1 *μ*M cisplatin, and brown line—cells treated with 2 *μ*M cisplatin. Both CCTL12 and CCTL14 lines of hESC were used (*n* = 2); a representative picture is shown.

The initiation and execution of apoptosis involves the employment of molecules in the cell membrane, cytoplasm, mitochondria, and cell nucleus. Typically, TRAIL launches the mitochondrial (intrinsic) apoptotic pathway via the activation of Bid, a Bcl-2 protein family member, by activated initiator caspase 8 and/or 10 [19, 20]. Other contributors to the regulation of the intrinsic pathway are the proapoptotic protein Bax and the antiapoptotic proteins XIAP, Bcl-xL, and Mcl-1. Here, we have determined the levels and/or activation status of all these regulators in cisplatin-treated hESC to possibly find their link to the TRAIL-sensitive cell phenotype. As shown in Figure 2(c), the exposure of hESC to cisplatin have led to the upregulation of inactive forms of both caspase 8 and caspase 10, while the expression of anti- and proapoptotic regulators remained unaffected (Figure 3(a)). It is notable that we have also found some minimal changes to the expression of the TRAIL receptors, specifically the weak upregulation of DR4, DR5, and DcR1, that may point to their contribution to development of TRAIL sensitivity (Figure 3(b)). Finally, we showed that hESC exposed to cisplatin retained their undifferentiated phenotype, as demonstrated by the high expression of pluripotency-associated membrane markers TRA-1-81 and SSEA-3 (Figure 3(c)). It is important information since DNA damage was previously shown to initiate the differentiation program in mouse embryonic stem cells [21].

### 3.4. Caspase 8 and Not Caspase 10 Mediates Apoptosis Induced by TRAIL in Cisplatin-Sensitized hESC

**Figure 4:**
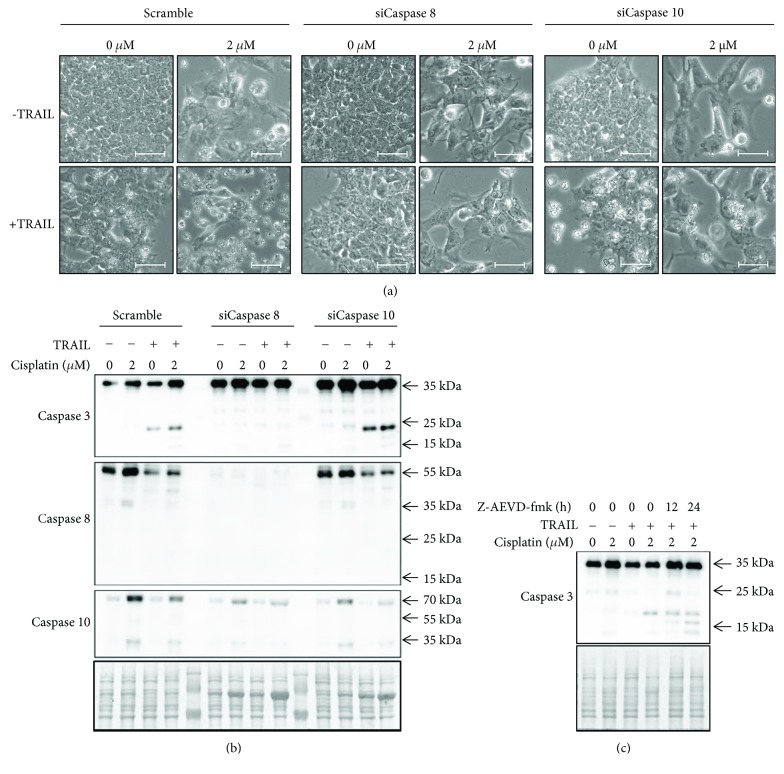
Downregulation of caspase 8 abolishes apoptosis induced by cisplatin and TRAIL. (a) Morphology of hESC upon siRNA-mediated knockdown of caspases 8 and 10, respectively, followed by combined cisplatin and TRAIL treatment, as observed by light microscopy (10x, bar 50 *μ*m). (b) Activation of the caspase cascade upon the given treatments are demonstrated by western blot visualization of the following inactive (full length) caspases: procaspase 3 (~35 kDa), procaspase 8 (~55 kDa), and procaspase 10 (~60 kDa). Their active forms include the following: cleaved caspase 3 (~17/12 kDa), cleaved caspase 8 (~18 kDa), and cleaved caspase 10 (~20 kDa). Staining with 0.1% amidoblack was used to evaluate the protein loading. Both CCTL12 and CCTL14 lines of hESC were used (*n* = 2); a representative picture is shown. To demonstrate caspase 8 activation under control (scrambled) conditions, a longer exposure of the membrane with the visible ~18 kDa fragment is shown in Supplementary Figure 3. (c) Western blot analysis of caspase 3 activation in hESC upon treatment with cisplatin, TRAIL, and caspase 10 inhibitor Z-AEVD-fmk (20 *μ*M). The CCTL14 line of hESC was used (*n* = 2).

The data above indicated that increased levels of caspase 8, caspase 10, and/or both, can be involved in the development of the sensitivity of hESC to TRAIL, induced here by cisplatin. Therefore, we have employed a siRNA-mediated knockdown strategy to investigate the role of these caspases in the initiation of TRAIL-induced apoptosis pathways in both pristine and cisplatin-treated hESC. As obvious from findings that are summarized in Figures 4(a) and 4(b), the downregulation of caspase 10 does not prevent the death of hESC and cleavage of their caspase 3. In contrast, hESC with downregulated caspase 8 do not die and their caspase 3 remains unprocessed. Indeed, incomplete silencing of caspase 10 may also be the reason for persistent dying, so that we also used an alternative strategy, chemical inhibition by the Z-AEVD-fmk inhibitor, to downregulate this enzyme. As shown in Figure 4(c), such chemical inhibition of caspase 10 produced the same outcome as appropriate siRNA, further documenting that only caspase 8 and not caspase 10 is indispensable for the initiation of apoptosis induced by TRAIL in sensitized hESC.

## 4. Discussion

Human embryonic stem cells are derivatives of the inner cell mass of the blastocyst that normally gives rise to all somatic cell types of the body. Given such developmental significance, hESC should expectably be equipped with a variety of mechanisms preventing the propagation of abnormalities to their genome and/or phenome. It is well known that upon stress stimuli, hESC can rapidly undergo apoptosis due to mitochondrial readiness—a shift in balance among the pro- and antiapoptotic proteins that favors proapoptotic signaling [5–7]. While intrinsic apoptotic signaling in hESC is well understood, not much is known about extrinsic receptor-dependent signaling in these cells. Our group has previously shown that although hESC express all the molecules required for the transduction of an extrinsic apoptotic signal, they are still resistant to TRAIL. We have also shown that this resistance is not absolute but can be overcome by proteosynthesis inhibitor homoharringtonine (HHT) [8, 22]. In the current study, we now demonstrate that hESC can be sensitized towards TRAIL-induced apoptosis also using the well-established DNA-damaging agent cisplatin. We provide evidence that cisplatin in concentrations of 1 *μ*M and 2 *μ*M causes DNA damage in hESC that can be repaired within 24 hours after cisplatin removal, without inducing excessive cell death. In this context, our results corroborate experiments performed on mouse ES cells (mESC), in which 8-hour treatment with 5 *μ*M cisplatin is considered as sublethal. Prolonged exposure of mESC to cisplatin then results in the induction of cell death [23–25].

It is notable that the morphology of hESC became altered upon treatment with cisplatin. This is in consonance with the findings made on various cancer cell lines, which dose-dependently change cell shape, increase the size of cell nuclei, and swell cell cytoplasm as a result of exposure to cisplatin [26–28]. It was also possible that the change of hESC morphology was associated with DNA-damage-induced entry into the differentiation pathway, as it has been previously shown in mESC [21]. Still, at 24 hours after cisplatin treatment we did not detect any changes to the expression of membrane markers that are strictly associated with pluripotency. Such findings are supported by the work of Wilson et al., who have demonstrated that strong ionizing radiation, causing DSB similar to cisplatin, also did not affect the levels of pluripotency markers in hESC [29].

Until this study, there was no report about the sensitizing of hESC to TRAIL by a DNA-damaging compound. Here, cisplatin treatment has led to a dramatic potentiation of TRAIL-mediated apoptosis, as on average ~78% of dead cells were detected after pretreating hESC with 1 *μ*M and 2 *μ*M doses of cisplatin. Enhanced activation of caspases 8 and 10 and processing of the effector caspase 3 and its substrate PARP were also detected, further confirming ongoing apoptosis. In terms of the capacity of hESC to acquire responsiveness to TRAIL, the new findings are congruent with the ones that we have previously made using HTT [8]. Still, the underlying mechanisms are principally different. While HHT promotes TRAIL sensitivity via the downregulation of antiapoptotic proteins Mcl-1 and cFLIP, cisplatin acts through increasing the expression of death receptors and initiator caspases. According to some authors, caspase 8 represents the central player that cannot be functionally substituted by caspase 10, whereas other works claim caspase 10 to be indispensable for TRAIL-mediated signaling [30–34]. Here, we provide evidence that caspase 8 and not caspase 10 is the central player mediating TRAIL-induced apoptosis in cisplatin-sensitized hESC.

Taken together, here we show that DNA damage, a stress stimulus to which hESC are highly sensitive, increases the capacity of hESC to activate the extrinsic apoptotic pathway. Our current as well as previous study collectively demonstrate that the molecular grounds underlying the sensitizing of hESC to TRAIL are analogous to such in cancer cells of TRAIL-resistant tumors [35–40]. We can now only speculate whether this is a characteristic feature of cells with a less-differentiated state represented here by hESC and such cells represented in tumors by “cancer stem/initiating cells.” Still, we are tempted to propose that this is in fact the case, so that development of sensitizing strategies on pluripotent stem cells (both embryonic and induced) and possibly also on adult stem cells, will contribute to establishing therapeutic scenarios for both regenerative medicine and oncology.

## 5. Conclusions

Here, we demonstrate that hESC, ex vivo derivatives of blastocyst stage embryos that are under normal conditions insensitive to TRAIL, become fully responsive to TRAIL after they transiently suffer from damage to their DNA caused by a crosslinking drug. This behavior is analogous to some cancer cells, which are also being grounded by analogous molecular mechanisms, specifically upregulation of TRAIL receptors and initiator caspases.

## Data Availability

The experimental data used to support the findings of this study are included within the article. Previously reported data were used to support this study and are available at doi: 10.1089/scd.2013.0057 and doi: 10.1111/febs.12347. These prior studies are cited at relevant places within the text as references [8, 18].

## Supplementary Materials

Supplementary Materials Supplementary Figure 1: flow cytometric analysis of apoptosis incidence upon treatment of hESC with cisplatin as determined by double staining with Annexin-V and propidium iodide. (a) Alteration to cell morphology upon the given treatment as demonstrated by a shift in the side scatter parameter. The *x* axis represents forward scatter, and the *y* axis represents side scatter. (b) Incidence of cell death upon the given treatment. The *x* axis represents Annexin-V, and the *y* axis represents propidium iodide. Quadrants: Q1 Annexin-/PI- (living cells), Q2 Annexin+/PI- (apoptotic cells), Q3 Annexin+/PI+ (secondary necrotic cells), and Q4 Annexin-/PI+ (necrotic cells). At least 10,000 cells were analyzed per sample. The CCTL14 line of hESC was used (*n* = 2). Supplementary Figure 2: flow cytometric analysis of apoptosis incidence upon combined treament of hESC with cisplatin and TRAIL as determined by double staining with Annexin-V and propidium iodide. (a) Alteration to cell morphology upon the given treatment as demonstrated by a shift in the side scatter parameter. The *x* axis represents forward scatter, and the *y* axis represents side scatter. (b) Incidence of cell death upon the given treatment. The *x* axis represents Annexin-V, and the *y* axis represents propidium iodide. Quadrants: Q1 Annexin-/PI- (living cells), Q2 Annexin+/PI- (apoptotic cells), Q3 Annexin+/PI+ (secondary necrotic cells), and Q4 Annexin-/PI+ (necrotic cells). At least 10,000 cells were analyzed per sample. The CCTL14 line of hESC was used (*n* = 2). Supplementary Figure 3: activation of caspase 8 upon the treatment of hESC with or without cisplatin and TRAIL, respectively, as demonstrated by western blot visualization. The picture represents a longer exposure of the membrane shown in Figure 4(b). The inactive full-length caspase at ~55 kDa and its active form at ~18 kDa are now visible where relevant.
