# Supplementary material for: Human Embryonic Stem Cells Acquire Responsiveness to TRAIL upon Exposure to Cisplatin
Source: Stem Cells Int. 2019 Jan 21;2019:4279481. doi: 10.1155/2019/4279481 (PMC6360567; doi:10.1155/2019/4279481)
Supplement: Supplementary Materials — Supplementary Figure 1: flow cytometric analysis of apoptosis incidence upon treatment of hESC with cisplatin as determined by double staining with Annexin-V and propidium iodide. (a) Alteration to cell morphology upon the given treatment as demonstrated by a shift in the side scatter parameter. The x axis represents forward scatter, and the y axis represents side scatter. (b) Incidence of cell death upon the given treatment. The x axis represents Annexin-V, and the y axis represents propidium iodide. Quadrants: Q1 Annexin-/PI- (living cells), Q2 Annexin+/PI- (apoptotic cells), Q3 Annexin+/PI+ (secondary necrotic cells), and Q4 Annexin-/PI+ (necrotic cells). At least 10,000 cells were analyzed per sample. The CCTL14 line of hESC was used (n = 2). Supplementary Figure 2: flow cytometric analysis of apoptosis incidence upon combined treament of hESC with cisplatin and TRAIL as determined by double staining with Annexin-V and propidium iodide. (a) Alteration to cell morphology upon the given treatment as demonstrated by a shift in the side scatter parameter. The x axis represents forward scatter, and the y axis represents side scatter. (b) Incidence of cell death upon the given treatment. The x axis represents Annexin-V, and the y axis represents propidium iodide. Quadrants: Q1 Annexin-/PI- (living cells), Q2 Annexin+/PI- (apoptotic cells), Q3 Annexin+/PI+ (secondary necrotic cells), and Q4 Annexin-/PI+ (necrotic cells). At least 10,000 cells were analyzed per sample. The CCTL14 line of hESC was used (n = 2). Supplementary Figure 3: activation of caspase 8 upon the treatment of hESC with or without cisplatin and TRAIL, respectively, as demonstrated by western blot visualization. The picture represents a longer exposure of the membrane shown in Figure 4(b). The inactive full-length caspase at ~55 kDa and its active form at ~18 kDa are now visible where relevant. [file 4279481.f1.pdf]

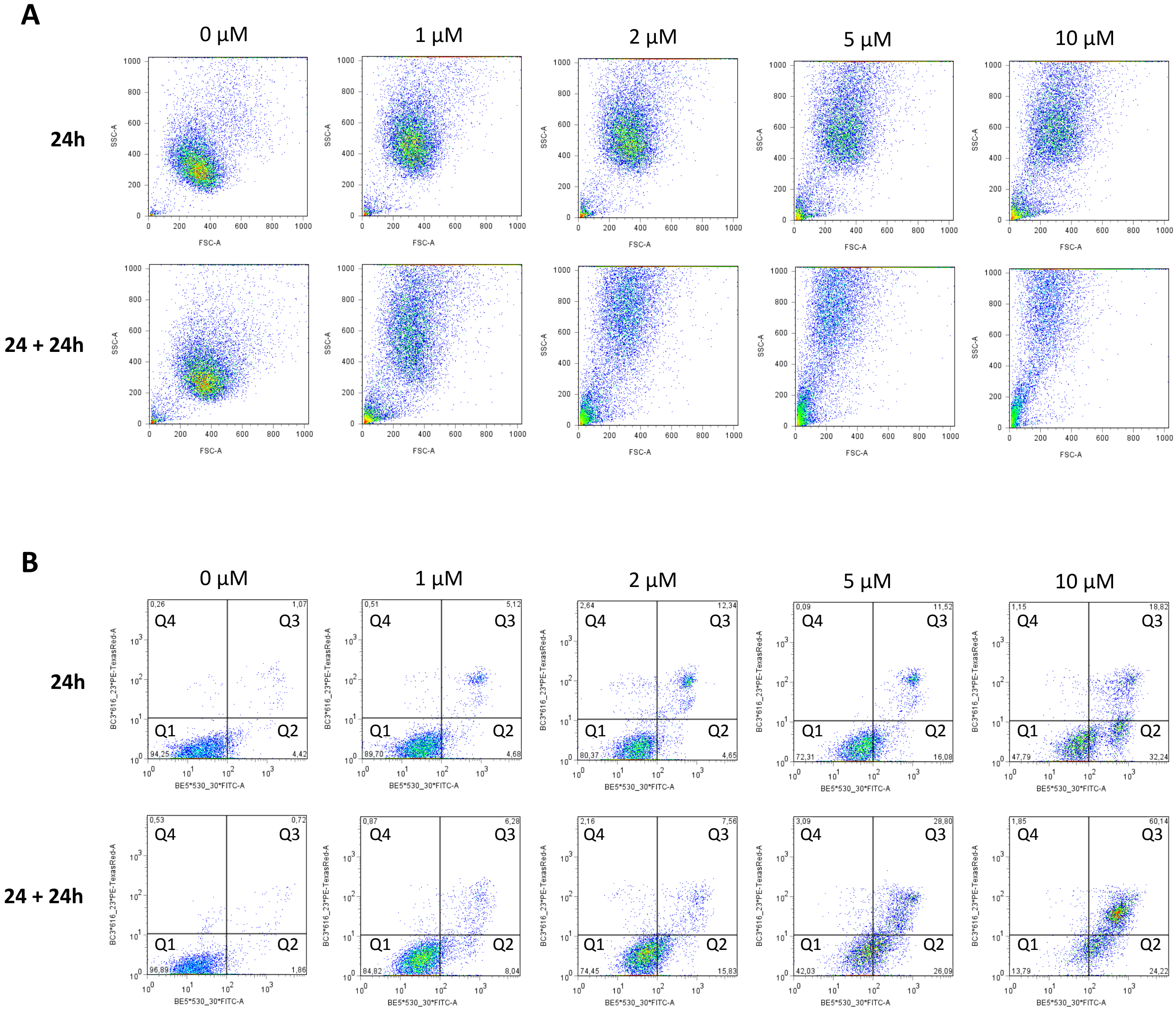

Supplementary Figure 1: Flow cytometric analysis of apoptosis incidence upon treatment of hESC with cisplatin as determined by double staining with Annexin-V and propidium iodide. **(A)** Alteration to cell morphology upon the given treatment as demonstrated by shift in side scatter parameter. X axis - forward scatter, y axis - side scatter. **(B)** Incidence of cell death upon the given treatment. X axis - Annexin-V, y axis - propidium iodide. Quadrants: Q1 Annexin-/PI- (living cells), Q2 Annexin+/PI- (apoptotic cells), Q3 Annexin+/PI+ (secondary necrotic cells), Q4 Annexin-/PI+ (necrotic cells). At least 10,000 cells were analyzed per sample. CCTL14 line of hESC was used, (n = 2).

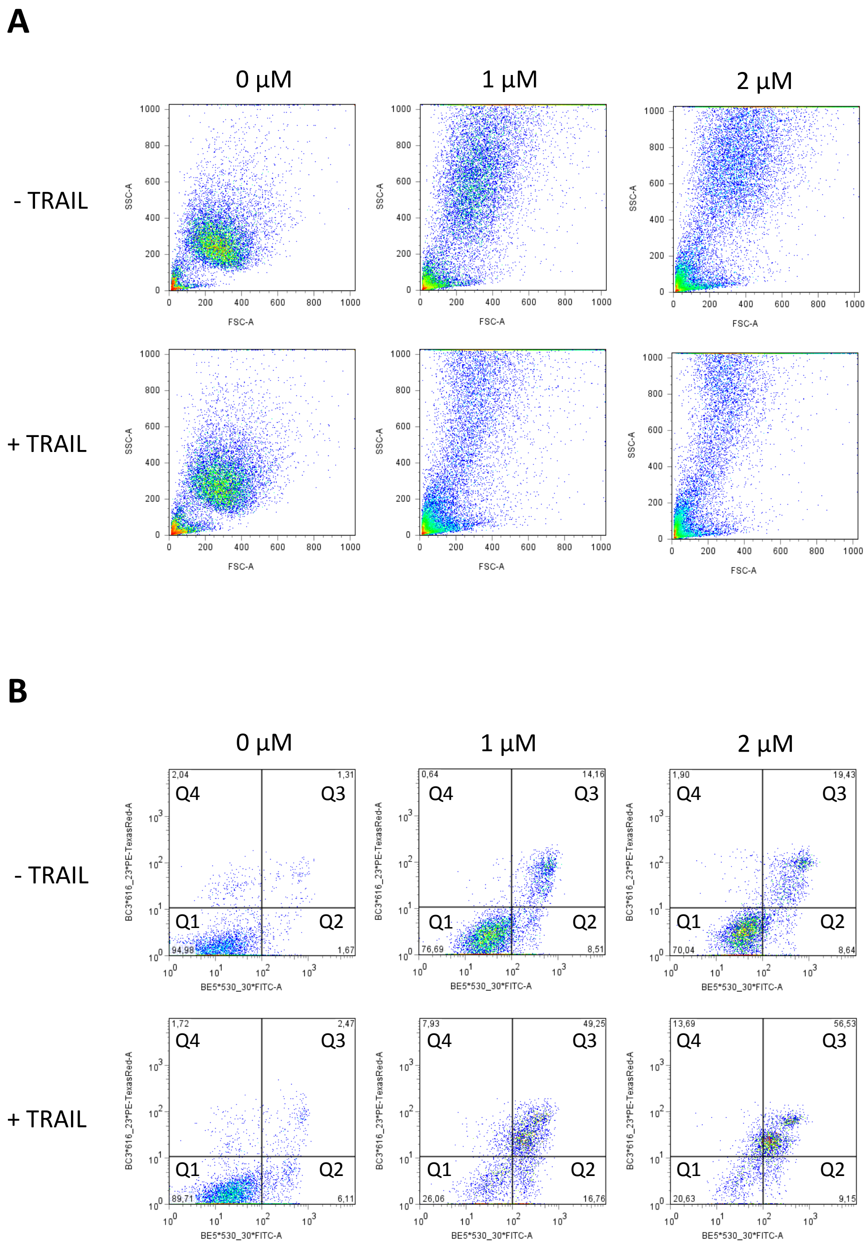

Supplementary Figure 2: Flow cytometric analysis of apoptosis incidence upon combined treament of hESC with cisplatin and TRAIL as determined by double staining with Annexin-V and propidium iodide. **(A)** Alteration to cell morphology upon the given treatment as demonstrated by shift in side scatter parameter. X axis - forward scatter, y axis - side scatter. **(B)** Incidence of cell death upon the given treatment. X axis - Annexin-V, y axis - propidium iodide. Quadrants: Q1 Annexin-/PI- (living cells), Q2 Annexin+/PI- (apoptotic cells), Q3 Annexin+/PI+ (secondary necrotic cells), Q4 Annexin-/PI+ (necrotic cells). At least 10,000 cells were analyzed per sample. CCTL14 line of hESC was used, (n = 2).

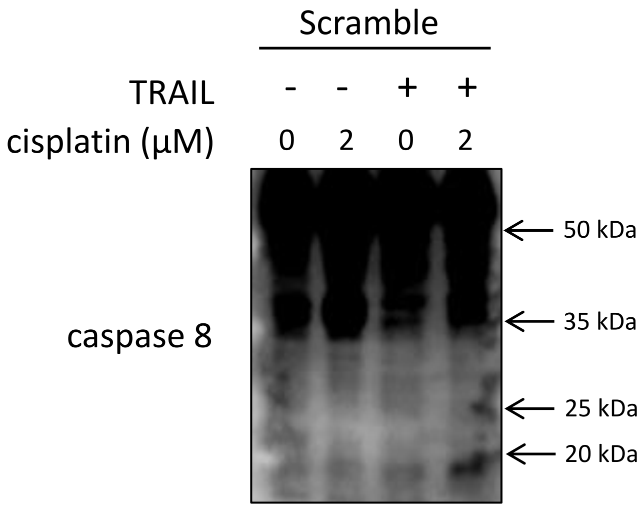

Supplementary Figure 3: Activation of caspase 8 upon the treatment of hESC with or without cisplatin and TRAIL, respectively, as demonstrated by western blot visualization. The picture represents a longer exposure of the membrane shown in Figure 4B. Inactive full length caspase ~ 55 kDa and its active form ~ 18 kDa are now visible where relevant.
